# DSTANet: A Lightweight and High-Precision Network for Fine-Grained and Early Identification of Maize Leaf Diseases in Field Environments

**DOI:** 10.3390/s25164954

**Published:** 2025-08-10

**Authors:** Xinyue Gao, Lili He, Yinchuan Liu, Jiaxin Wu, Yuying Cao, Shoutian Dong, Yinjiang Jia

**Affiliations:** 1 College of Electrical Engineering and Information, Northeast Agricultural University, Harbin 150030, China; xinyuegao@neau.edu.cn (X.G.);; 2 Key Laboratory of Northeast Smart Agricultural Technology, Ministry of Agriculture and Rural Affairs, Harbin 150030, China; 3 Department of Academic Theory Research, Northeast Agricultural University, Harbin 150030, China

**Keywords:** maize diseases identification, image classification, feature extraction, DSTANet

## Abstract

Early and accurate identification of maize diseases is crucial for ensuring sustainable agricultural development. However, existing maize disease identification models face challenges including high inter-class similarity, intra-class variability, and limited capability in identifying early-stage symptoms. To address these limitations, we proposed DSTANet (decomposed spatial token aggregation network), a lightweight and high-performance model for maize leaf disease identification. In this study, we constructed a comprehensive maize leaf image dataset comprising six common disease types and healthy samples, with early and late stages of northern leaf blight and eyespot specifically differentiated. DSTANet employed MobileViT as the backbone architecture, combining the advantages of CNNs for local feature extraction with transformers for global feature modeling. To enhance lesion localization and mitigate interference from complex field backgrounds, DSFM (decomposed spatial fusion module) was introduced. Additionally, the MSTA (multi-scale token aggregator) was designed to leverage hidden-layer feature channels more effectively, improving information flow and preventing gradient vanishing. Experimental results showed that DSTANet achieved an accuracy of 96.11%, precision of 96.17%, recall of 96.11%, and F1-score of 96.14%. With only 1.9M parameters, 0.6 GFLOPs (floating point operations), and an inference speed of 170 images per second, the model meets real-time deployment requirements on edge devices. This study provided a novel and practical approach for fine-grained and early-stage maize disease identification, offering technical support for smart agriculture and precision crop management.

## 1. Introduction

Maize is a widely cultivated crop, serving not only as food but also as industrial raw material with extensive applications in many fields. Current maize cultivation practices often involve extensive and continuous monoculture, which could result in the rapid spread of diseases, leading to huge economic losses [1]. In addition, the overuse of pesticides pollutes the field environment, reduces soil fertility, enhances the resistance of pathogens, and threatens the sustainability of maize cultivation.

Traditional methods rely on manual identification based on the experience of agricultural experts. Jurado et al. (2006) [2] proposed a polymerase chain reaction detection method to identify pathogenic fusarium in maize. But these traditional methods are subjective and can identify diseases only when symptoms are significant, making the timely identification of early-stage diseases challenging. Kusumo et al. (2018) [3] and Panigrahi et al. (2020) [4] used some machine learning algorithms to evaluate the effect of various image features in maize disease detection. The introduction of machine learning methods has enabled the automatic identification of diseases, but there is still a problem that it is difficult to extract disease features automatically, and feature extractors also need to be designed manually.

With the development of deep learning and computer vision, fine-grained image identification technology has achieved significant breakthroughs. Representative algorithms include ResNet50 [5] and vision transformer [6]. Building upon these algorithms, researchers have made improvements to meet the needs of the automatic identification of crop diseases. Yang et al. (2023) [7] proposed re-GoogLeNet for the accurate identification of rice leaf diseases in field environments, enhancing the feature extraction capability for small, irregularly shaped spots on diseased leaves. Zhang et al. (2023) [8] introduced a progressive non-local means algorithm to solve the challenge of inter-class similarity and intra-class variability in tomato leaf diseases, and introduced a multi-channel automatic directional recurrent attention network. Liu et al. (2024) [9] constructed the GLDCNet for identifying grapevine leaf roll diseases based on UAV RGB images, achieving an accuracy of 99.57%. Liu et al. (2024) [10] constructed a Multi-scale constrained MCDCNet based on multi-branch convolution and deformable convolution to identify apple leaf diseases. Compared to SOTA models, it improved accuracy by 3.85% and could accurately classify five common apple leaf diseases. Zhang et al. (2024) [11] combined leaf vein features with other textural features to generate high-quality semantic features. They designed a Multi-Attention IBN Anti-aliasing Network based on Fourier analysis for identifying cassava leaf diseases.

At the same time, deep learning algorithms have also been widely applied to the field environment of maize leaf disease identification. Zeng et al. (2022) [12] employed a lightweight dense-scale network to improve the identification accuracy of maize diseases. Addressing the challenges of small sample sizes and complex backgrounds, Li et al. (2023) [13] applied ACGAN to augment the maize diseases dataset. By combining this with transfer learning, they established a practical method for identifying maize leaf diseases under field conditions. Xu et al. (2023) [14], building on ResNet50, introduced the ECA attention mechanism and the Adam optimizer to identify six types of maize diseases and pests, achieving an identification accuracy of 93.95%. Bai et al. (2024) [15] found that the use of hyperspectral images could better understand the response of maize plants to leaf spot disease infection, which was conducive to improving the early detection strategy. Wang et al. (2024) [16] proposed a texture-color dual-branch multi-scale residual shrinkage network (TC-MRSN). One branch of this network uses the improved LBP algorithm to extract texture features, and the other branch utilizes the RGB features of the convolutional neural network, retaining the feature information of small lesions. The results showed that the optimized model achieved an identification accuracy of 94.88%. Zhang et al. (2024) [17] introduced separable convolutions and attention mechanisms to enhance the extraction capability of maize disease features, proposing LSANNet for maize leaf disease identification, and the accuracy achieved to 94.35%. In order to reduce unnecessary redundant spatial information, Wang et al. (2024) [18] used octave convolution to accelerate training. They established OSCRNet to achieve the interaction of different feature information within images. Li et al. (2025) [19] integrated high-frequency detail information in multiple layers of MobileNetV2, using HFFE (high-frequency feature extraction) to enhance the network’s ability to learn detail information, achieving an accuracy of 95.7%. To reduce the semantic ambiguity on RGB single-modal data and enhance the connection between images and text, Wang et al. (2025) [20] proposed WCG-VMamba, which utilized multi-modal data of text and images to identify four common maize diseases, improving the recognition accuracy in complex environments, achieving an accuracy of 99.23%. Based on the hyperspectral maize disease imagery, Liu et al. (2024) [21] proposed an attention-based spatial-spectral joint network, which enhanced the model’s identification capabilities by extracting features from spatial and spectral dimensions. Compared to traditional disease identification methods based on image classification, object detection algorithms such as YOLO enable the precise detection and localization of disease lesions [22]. For instance, studies by Yang et al. (2024) [23] and Li et al. (2024) [24] both achieved high-precision identification of maize diseases by improving the YOLOv8. However, this approach is highly dependent on accurately annotated datasets. Furthermore, the bounding box regression process incurs significant computational overhead, which increases the model’s training costs and poses challenges for deployment on edge devices.

However, in the field environment, the inter-class similarity and intra-class variability of maize diseases brought a huge challenge to precise identification. Certain different types of maize diseases exhibited similar symptoms, which could lead to error if identification only relied on the color and shape features of the lesions. For instance, both gray leaf spot [25] and late-stage northern leaf blight [26] manifested as tan-brown stripes. Similarly, common rust [27] and eyespot [28] often presented as small, yellowish-brown, spot-like lesions. In addition, the morphology of the same diseases was variable in different stages. There were only sporadic disease spots in the early stage of eyespot, while the infected leaves would wither in the late stage. Therefore, identifying different maize leaf diseases in time and correctly was of vital importance in agricultural production to avoid economic losses caused by disease flooding.

In addition, most studies relied on public datasets with simple backgrounds and lacked early-stage disease identification. First, early-stage lesions were extremely small, often comprising only a few pixels in size, making them easily overlooked. Second, early disease symptoms exhibited subtle color differences from healthy leaf tissue, typically presenting slight yellow-green variations that were difficult to distinguish from natural color variations in leaves. Additionally, early symptoms have irregular morphology with blurred boundaries and lack distinct characteristic markers, which pose significant challenges for automated recognition algorithms. Environmental factors also affect the accuracy of early detection, as changes in lighting conditions can alter the reflective properties of leaf surfaces, further increasing identification difficulty. And identifying early diseases in time can reduce pesticide use, achieve targeted spraying, avoid environmental pollution, and promote sustainable agricultural development. These challenges underlined the practical importance of developing precise identification methods specifically for early-stage diseases.

To deal with the above-mentioned challenges, the main contributions of this study are as follows:

(1) To address the inter-class similarity and intra-class variability characteristics of maize leaf diseases, the decomposition spatial fusion module (DSFM) was constructed, which could accurately locate the lesions on the leaves and overcome the influence of redundant environmental noise.
(2) The multi-scale token aggregator (MSTA) was introduced, which utilized depthwise separable convolution at different scales to achieve the fusion and complementation of feature information, thereby obtaining more complete and comprehensive feature information.
(3) Combining DSFM and MSTA, this study proposed a decomposed spatial token aggregation network (DSTANet) that integrated the advantages of local feature extraction of CNN and global feature extraction of transformer and had the ability to identify early-stage maize diseases.
(4) A dataset comprising six different types of maize leaf diseases and healthy leaves was created. Data for both early and late stages of eyespot and northern leaf blight were collected. This dataset served as the basis for validating the superiority of DSTANet.

## 2. Materials and Methods

### 2.1. Materials

**Figure 1:**
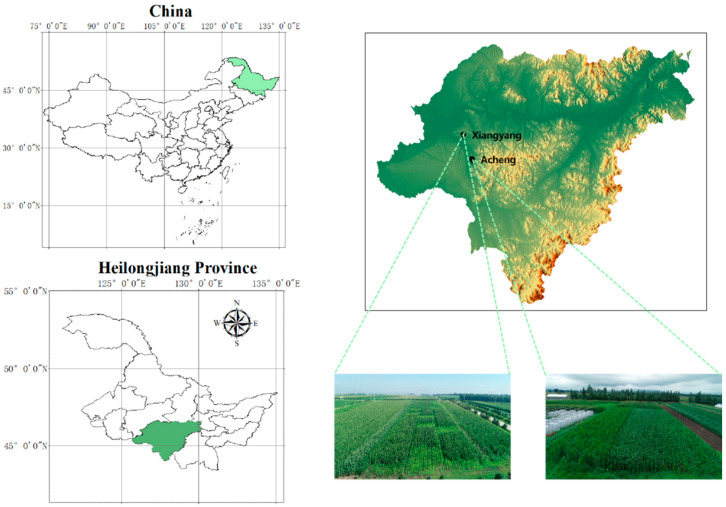
Geographical location of the experimental areas.

The maize disease images in this study were collected from Xiangyang Farm and Acheng Farm, located in Harbin City, China. The geographical information of the experimental area is shown in Figure 1. And the data acquisition protocol is shown in Table 1.

**Figure 2:**
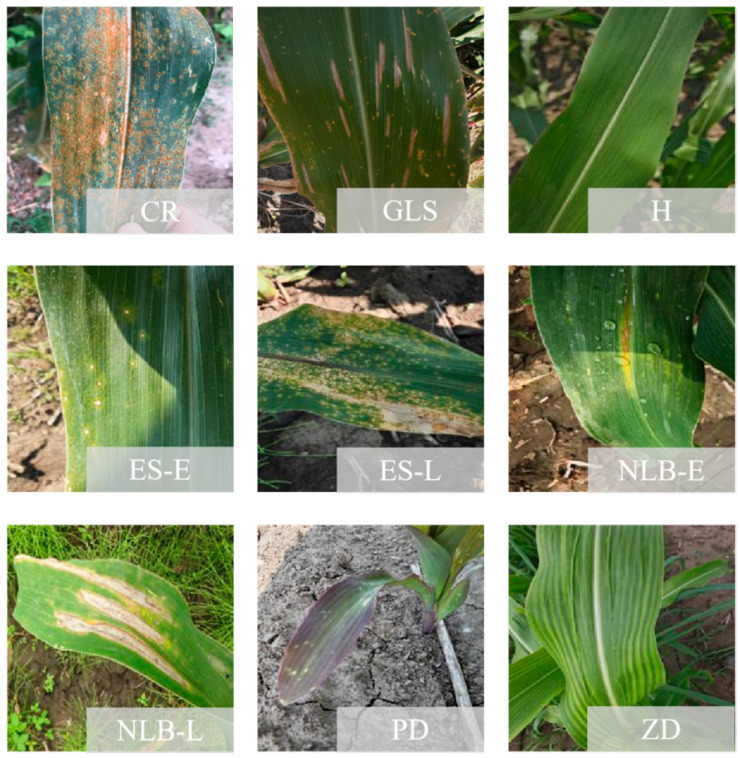
Example images of maize leaf diseases.

We expanded the data samples by mixing self-built datasets and public datasets. Finally, healthy (H) leaves, early-stage eyespot (ES-E), late-stage eyespot (ES-L), early-stage northern leaf blight (NNB-E), late-stage northern leaf blight (NLB-L), phosphorus deficiency (PD), zinc deficiency (ZD), common rust (CR), and gray leaf spot (GLS) have 1000 images each, for a total of 9000 images. The public datasets were PlantVillage [29] and PlantDoc [30], which were publicly available on Kaggle (https://www.kaggle.com/). Examples of the maize diseases data were shown in Figure 2.

The original images were resized to 224 × 224 pixels. The dataset was subsequently split randomly into training, validation, and test sets at a ratio of 6:2:2. In order to evaluate the model’s performance more objectively, we applied data augmentation techniques, including random brightness adjustment (increase and decrease), salt-and-pepper noise addition, random erasing, and random scaling to the images in the training sets.

### 2.2. Methodologies

#### 2.2.1. Overall Structure of the Model

**Figure 3:**
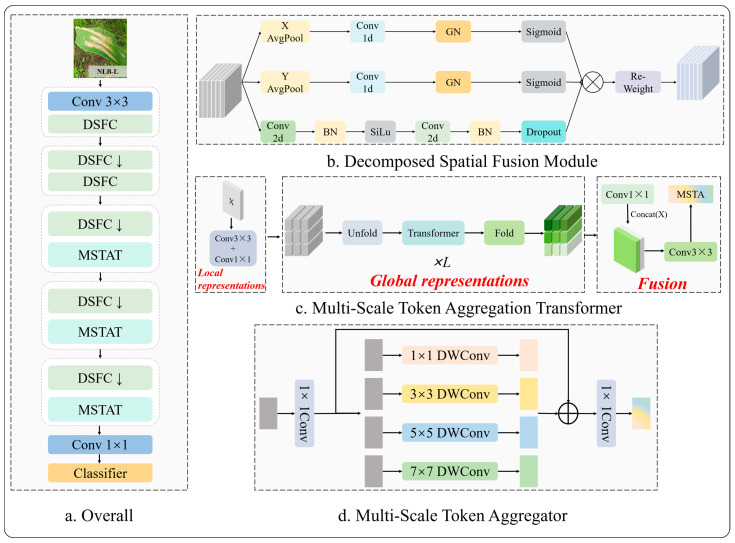
Overall model architecture: (**a**) Internal structure of DSTANet (The arrow represents downsampling). (**b**) Internal structure of decomposed spatial pusion module. (**c**) Internal structure of multi-scale token aggregation transformer. (**d**) Internal structure of multi-scale token aggregator.

The similarity between different maize leaf diseases, the variability within the same disease across different stages, and the complexity of the real field environment lead to challenges for existing disease identification models in practical applications, including poor generalization and low accuracy. In order to address these problems, this study introduced a multi-stage neural network DSTANet that combined CNNs and transformers. This model took MobileViT [31] as the baseline and combined DSFM and MSTA. The model’s overall architectural framework is depicted in Figure 3a.

#### 2.2.2. Decomposed Spatial Fusion Module

During feature extraction, attending to both inter-channel relationships and spatial positional relationships within feature maps could enable models to achieve better results. Classic attention mechanisms such as SENet [32] and CBAM [33] primarily considered encoding information based on inter-channel relationships, neglecting positional information. To effectively extract features of subtle lesions and mitigate the impact of inter-class similarity and intra-class variability for high-accuracy identification among maize leaf diseases, this study was inspired by ELA [34] and integrated one-dimensional convolution and group normalization [35] for feature enhancement. This method constructed a multi-branch feature extraction module, named decomposed spatial fusion module (DSFM). In the first and second branches, two one-dimensional positional feature maps were encoded to enhance the extraction capabilities for ambiguous and subtle features, capturing global contextual information. The third branch performed convolution and pooling operations for deeper processing and learning of more abstract local information features. The structure of DSFM was illustrated in Figure 3b.

Firstly, the input $X\in R^{C\times H\times W}$ is subjected to average pooling along the horizontal and vertical directions to capture long-range dependencies, mitigating the influence of irrelevant regions on predicting the category of the diseases. The formula is represented as follows, where $x_{c}$ denotes the information of a single channel feature map, $X_{c}^{h}\left(h\right)$ and $X_{c}^{w}\left(w\right)$ capture the global receptive field and precisely locate the significant features of the diseases.

(1) $$X_{c}^{h}\left(h\right)=\frac{1}{W}\sum_{0\leq i<W}x_{c}\left(h,i\right)$$

(2) $$X_{c}^{w}\left(w\right)=\frac{1}{H}\sum_{0\leq j<H}x_{c}\left(j,w\right)$$

The coordinate information obtained from the above formulas is input into a 1D convolution to enhance the positional encoding weights in the horizontal and vertical directions. Compared to 2D convolution, 1D convolution is not only adept at handling sequential information but is also more lightweight with higher computational efficiency. To precisely locate regions of interest, the DSFM employs 1D convolution with a kernel size of 7, which decides the coverage range of local interactions. The enhanced coordinate information is subsequently processed through group normalization, producing positional attention representations in both horizontal and vertical directions. The formulas are as follows.

(3) $$y^{h}=GN\left({Conv1d}_{h}\left(X_{c}^{h}\left(h\right)\right)\right)$$

(4) $$y^{w}=GN\left({Conv1d}_{w}\left(X_{c}^{w}\left(h\right)\right)\right)$$

The third branch employs a 3 × 3 convolution to expand the input X, thereby acquiring richer feature information, which is computed as $y^{ep}\in R^{H\times W\times (C\times ep)}$. Subsequently, the pointwise convolution maps the feature channels to a higher dimension, and the dropout layer is introduced to prevent co-adaptation and overfitting within the model, yielding $y^{pro}\in R^{H\times W\times D}$, where D≥C.

(5) $$y^{ep}=expand\text{\_}conv(X)$$

(6) $$y^{pro}=project\text{\_}conv(y^{ep})$$

Finally, the feature tensor on the three parallel branches is fused and the weights is reallocated to obtain Y. The re-weight module consists of depthwise separable convolution, average pool, and a nonlinear activation function σ. The formula is as follows:

(7) $$Y=\sigma (AvgPool(DWConv2d(y^{h}*y^{w}*y^{pro})))$$

#### 2.2.3. Multi-Scale Token Aggregator

Traditional vision transformers lacked the local inductive bias characteristic of convolutional neural networks. MobileViT addressed this limitation by integrating the locality of CNNs with the globality of ViT. However, a single-scale token aggregation mechanism could not fully leverage the abundant feature channel information within the hidden layers [36]. Therefore, we introduced the multi-scale token aggregator (MSTA). As shown in Figure 3c, the core of the module is a multi-scale aggregation mechanism, which processes and fuses features across different scales, depthwise separable convolutions in parallel. This enhances the model’s ability to perceive disease spots of various sizes in leaf disease images. Meanwhile, the application of depthwise separable convolutions effectively reduced the model’s computational overhead, thus satisfying the lightweight requirements.

Firstly, the input feature map $X\in R^{H\times W\times C}$ is mapped to a higher dimension via a pointwise convolution to obtain $X_{H}\in R^{H\times W\times \left(C\times e\right)}$, where e is the channel expansion factor. This convolution layer consists of a 1 × 1 convolution, a GELU activation function, and a batch normalization layer. Subsequently, the $X_{H}$ is processed in parallel by four different depthwise separable convolutions, with each convolution operating on one-quarter of the channels, resulting in $X_{1}$, $X_{2}$, $X_{3}$, $X_{4}$. Convolutions with kernel sizes of 3, 5, and 7 effectively capture multi-scale information, while the 1 × 1 convolution acts as a learnable channel dimension expansion factor. And the formulas are as follows:

(8) $$X_{H}={BN(GELU(Conv}_{1\times 1}(X)))$$

(9) $$\left\{\begin{array}{l} {X_{1}=DWConv}_{1\times 1}\left(X_{\frac{H}{4}}\right)\\ X_{2}={DWConv}_{3\times 3}\left(X_{\frac{H}{4}}\right)\\ X_{3}={DWConv}_{5\times 5}\left(X_{\frac{H}{4}}\right)\\ X_{4}={DWConv}_{7\times 7}\left(X_{\frac{H}{4}}\right) \end{array}\right.$$

Finally, the output of the multi-scale depthwise separable convolution is added to the original features through the residual connection. This facilitates information flow, avoids vanishing gradients, and enables information fusion and complementarity between features at different scales. And another 1 × 1 point convolution is used for channel dimension reduction, and the feature map is restored to the original input dimension to obtain the final output $X_{out}\in R^{H\times W\times C}$. The formula is as follows:

(10) $$X_{out}={(Conv}_{1\times 1}(Contact\left(X_{1},X_{2},X_{3},X_{4}\right)+X_{H}))$$

#### 2.2.4. Multi-Scale Token Aggregation Transformer

The multi-scale token aggregation transformer (MSTAT) is composed of three modules: local information encoder, global information encoder, and multi-scale feature fusion unit. The specific implementation is shown in Figure 3d.

Firstly, the local information encoder consists of a 3 × 3 convolution and a 1 × 1 convolution. The 3 × 3 convolution is used for local feature encoding, while the 1 × 1 pointwise convolution maps the feature map to a higher-dimensional feature space, resulting in the output $X_{L}$.

(11) $$X_{L}={Conv}_{1\times 1}\left({Conv}_{3\times 3}\left(X\right)\right)$$

Secondly, to enable the model to learn global information with spatial inductive bias, in the transformer-based global information encoder, $X_{L}$ is unfolded into N non-overlapping patches of equal size to obtain $X_{U}\in R^{P\times N\times d}$ (where P=h×w, $N=\frac{HW}{P}$, P represents the number of pixels in each patch, N represents the number of patches, and h<n, w<n are the height and width of each patch, respectively). $X_{U}$ is input into L stacked Transformer layers to encode global information and obtain the dependencies between patches, resulting in $X_{G}\in R^{P\times N\times d}$. The formulas are as follows:

(12) $$X_{U}=Unfold\left(X_{L}\right)$$

(13) $$X_{G}\left(p\right)=Transformer\left(X_{U}\left(p\right)\right),1\leq p\leq P$$

Since $X_{U}(p)$ employs 3 × 3 convolution for local information encoding, and $X_{G}(p)$ encodes the global information for the p-th location across P patches, each weight value in $X_{G}$ represents an encoding of information from all pixels in X. Therefore, the overall effective receptive field of the MSTAT is H × W [31].

Unlike traditional vision transformers that discard inherent spatial relationships (both between patches and within each patch), this module retains hierarchical structure at both levels. Specifically, it preserves the topological order of patches across the image while maintaining the absolute spatial arrangement of pixels within every local patch, thus capturing fine-grained positional information lost in standard ViT architectures. So $X_{G}$ is folded to obtain $X_{F}\in R^{H\times W\times d}$.

(14) $$X_{F}=Fold\left(X_{G}\right)$$

In the multi-scale feature fusion unit, the MSTAT employs a 1 × 1 pointwise convolution to project $X_{F}$ into a C-dimensional space, and then concatenates it with the original input X via a concatenation operation to obtain $X_{Fm}$. Subsequently, a 3 × 3 convolution is applied to $X_{Fm}$ for preliminary feature fusion, and the result is then fed into the MSTA for multi-scale feature aggregation to obtain Y.

(15) $$X_{Fm}=contact\left(X,{Conv}_{1\times 1}\left(X_{F}\right)\right)$$

(16) $$Y=MSTA\left({Conv}_{3\times 3}\left(X_{Fm}\right)\right)$$

## 3. Results

### 3.1. Evaluation Index

In order to evaluate the effectiveness of the proposed model in identifying maize leaf diseases, this study used accuracy, precision, recall, and F1-score as evaluation indicators.

(17) $$Accuracy=\frac{TP+TN}{TP+TN+FP+FN}$$

(18) $$Precision=\frac{TP}{TP+FP}$$

(19) $$Recall=\frac{TP}{TP+FN}$$

(20) $$F1-score=\frac{2\times Precision\times Recall}{Precision+Recall}$$

TP represents the instance for which both predicted and actual values are positive. FP represents the instance for which the predicted value is positive but the actual value is negative. FN represents the instance for which the predicted value is negative but the actual value is positive. TN represents the instance for which both predicted and actual values are negative. Accuracy is the proportion of all correctly classified samples to the total number of samples. Precision is the proportion of samples predicted as positive that were actually correct, reflecting the purity of the model’s positive predictions. Recall is the proportion of all actual positive samples that the model successfully identified, reflecting the model’s ability to find all relevant instances. F1-score provides a balanced measure between precision and recall by calculating their harmonic mean. To provide a comprehensive evaluation across all classes, the Macro Average was used.

### 3.2. Parameter Setting

All the model training and testing in this study were deployed on the NVIDIA GeForce RTX 3090 24GB machine (NVIDIA, Santa Clara, CA, USA). The software environment was based on Python 3.8 and PyTorch 1.8.1. Other configurations included: the operating system was Ubuntu 20.04, the CPU was an AMD EPYC 7642, the RAM was 80 GB, and the GPU computing platform was CUDA 11.1.

Learning rate and optimizer are indispensable hyperparameters in the model training process, which have a direct influence on the model learning process and final performance. And the initial learning rate determines the pace of weight update in each iteration. And the optimizer is used to guide how to update the model weights based on the loss function.

Under the premise that the learning rate was set as 0.001, the influence of SGD [37], Adam [38], and AdamW [39] on the accuracy of DSTANet was compared. The specific results were shown in Table 2, where the model obtained the best result when AdamW was selected. To systematically evaluate the impact of different learning rates on DSTANet’s recognition accuracy, we employed the AdamW optimizer across all experiments. The results are shown in Table 3, where the model performed best when the initial learning rate was 0.001. This study explored the dynamic optimization path for model training by fine-tuning the optimizer configurations and learning rate. Final hyperparameter selection converged on AdamW with a 0.001 learning rate, balancing convergence speed and recognition accuracy.

According to the above experimental results, the parameter settings for the initial learning rate, batch size, epochs, optimizer, learning rate adjustment strategy, and loss function during the training process are shown in Table 4.

The experimental content of this study included the analysis of the effectiveness of DSTANet architecture, the comparison with different models, the performance analysis of different models, the analysis of similar diseases identification, and the ablation experiments. The confusion matrix and Grad-CAM [40] feature visualization tools were combined to verify the effectiveness and novelty of the model.

### 3.3. Analysis of the Effectiveness of DSTANet Architecture

To validate the effectiveness of the model architecture proposed in this study and to ensure the stability of the results, a 5-fold cross-validation method was employed. The dataset was divided into five equal folds while preserving the original class distribution. In each iteration, four folds were used for training, and the remaining one fold was used for validation. This process was repeated five times, allowing each fold to serve as the validation set once, thereby effectively minimizing bias and variance. The experimental results for DSTANet were presented in Table 5.

The accuracy for each fold exceeded 95%, which provided strong evidence for the effectiveness of the model architecture constructed in this study. Through cross-validation, the phenomenon of obtaining an accidentally high accuracy due to a random data split was mitigated.

### 3.4. Comparison with Different Models

To validate the superior identification performance of DSTANet for maize diseases in complex field environments, this section compares DSTANet with eight mainstream models. We selected representative state-of-the-art (SOTA) models in the field of computer vision in recent years, including various transformer-based network models such as MobileViT, DaViT [41], ViT, SwinTransformer [42], and traditional convolution neural network (CNN) models such as MobileNetV3 [43], EfficientNetV2 [44], ConvNeXt [45]. Additionally, we compared the currently popular VMamba [46] with our method.

**Figure 4:**
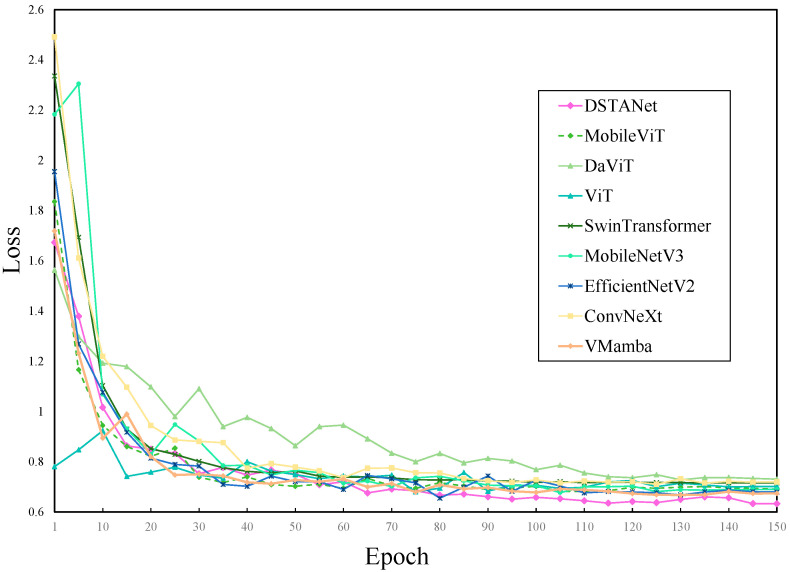
Comparison of loss of different models.

**Figure 5:**
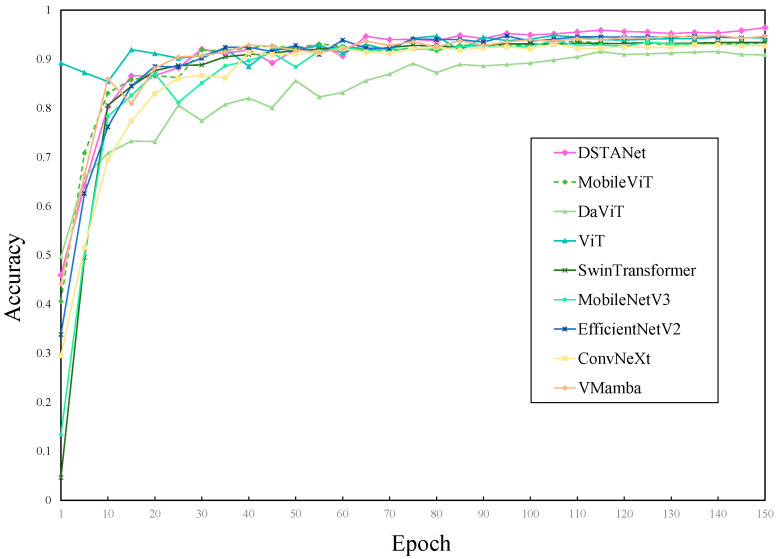
Comparison of accuracy of different models.

The results of different models based on the training set were shown in Figure 4 and Figure 5, where the model accuracy gradually increased with the increase of training epochs until the model was fitted. The loss value on the y-axis in Figure 4 represented the difference between the predicted label and the actual label. A larger loss value indicated a greater difference between the predicted result of the model and the actual result. From the training loss curve, it could be seen that the loss of DSTANet was smaller, decreased faster, and fluctuated the least during training. As shown in Figure 5, after the 65th epoch, DSTANet showed a faster convergence speed and higher accuracy than other models. After the 120th epoch of training, the accuracy of DSTANet gradually stabilized above 95%. The results of different models are shown in Table 6. DSTANet’s accuracy, precision, recall, and F1-score were 96.11%, 96.17%, 96.11%, and 96.14%, respectively, with all metrics being higher than those of other advanced disease identification models. Compared to traditional convolutional neural networks, DSTANet’s accuracy was 3.17% higher than that of the best-performing EfficientNetV2. In comparison with various vision transformer models, DSTANet’s accuracy was 4.61% higher than that of the best-performing SwinTransformer. And compared to VMamba, which was the most popular computer vision model, DSTANet’s accuracy was 3.33% higher.

### 3.5. Performance Analysis of Different Models

When designing and selecting a classification model, the parameters, floating point operations (FLOPs), and frames per second (FPS) are the indispensable evaluation metrics of the performance. Table 7 presents the comparison of parameters, FLOPs, and FPS for DSTANet compared with other models.

The parameters and FLOPs of DSTANet were only marginally higher than MobileViT and significantly lower than other models, achieving 1.9M and 0.6G. This was because the influence of the complex field environment was comprehensively considered during the design of the DSTANet structure. Therefore, DSTANet showed a higher scene fit. We measured the processing speed of each model for maize disease images under the same hardware environment. DSTANet achieved an FPS of 170, indicating its rapid response capability in practical deployments. Although some models have faster inference speed than DSTANet, DSTANet is still the best choice when considering identification accuracy and computational complexity. These results collectively demonstrated that DSTANet maintained high performance while possessing low model complexity and fast inference speed, making it suitable for deployment on hardware devices with limited resources for real-time applications.

### 3.6. Analysis of Inter-Class Similarity and Intra-Class Variability

**Figure 6:**
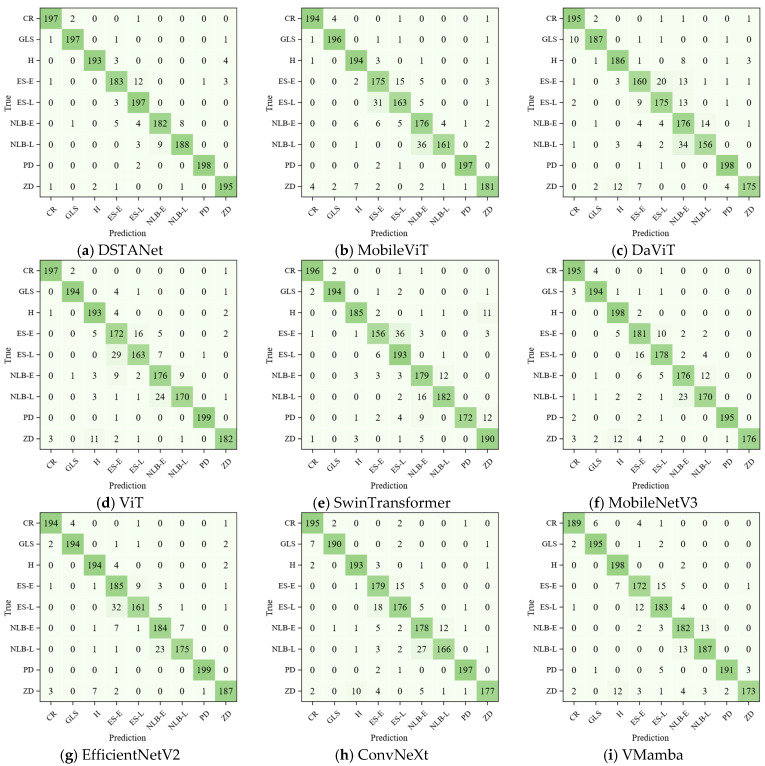
Confusion matrix of maize disease classification.

One of the most significant challenges in automated maize leaf disease diagnosis lies in accurately distinguishing between morphologically similar diseases that share comparable visual characteristics while managing the inherent variability within each disease category. In real-world agricultural scenarios, corn leaf diseases often exhibit high inter-class similarity, where different diseases present similar symptoms, similar coloration patterns, and comparable lesion morphologies. Simultaneously, some diseases demonstrate intra-class variability due to disease progression stages. To provide a more intuitive demonstration of DSTANet’s superior identification capability in a field environment compared to other models, Figure 6 shows the confusion matrices plotted based on each model.

Analysis of the confusion matrix revealed that DSTANet confused eyespot with common rust in only two instances and could accurately distinguish between late-stage northern leaf blight and gray leaf spot. Regarding the different stages of northern leaf blight, DSTANet had only 17 confusion instances, which was also lower than other models. For the different stages of eyespot, DSTANet misclassified only 15 instances between them, but the confusion rate between these stages in other models was significantly higher than DSTANet’s. Compared with DSTANet, although the baseline model MobileViT misclassified CR into ES-L in only one instance, it could also accurately distinguish late-stage northern leaf blight and gray leaf spot. However, for different stages of northern leaf blight, MobileViT had 40 confusions, far more than DSTANet (only 17). For different stages of the eyespot, MobileViT misclassified 46 instances. It could be seen clearly that the overall performance of MobileViT was worse than that of DSTANet.

These results demonstrated DSTANet’s robustness to both inter-class similarity and intra-class variability, enabling high accuracy in identifying little lesion signatures. By extending the transformer’s advantage to capture global features, it also solves the problem of small-scale feature disappearance caused by multiple convolutions.

The detailed identification results of DSTANet for each disease category are presented in Table 8. Out of the total 1800 images in the test set, DSTANet correctly classified 1730 samples. Its performance was significantly superior to other models.

### 3.7. Ablation Experiments

#### 3.7.1. Ablation Experiment of MSTA

To validate the effectiveness of the 4-branch design in MSTA, this section conducted comprehensive ablation experiments to evaluate the impact of different branch configurations on model performance. This study compared four different MSTA variants: a 2-branch version using 1 × 1 and 3 × 3 depthwise convolutions with 1/2 channel allocation each, a 3-branch version incorporating 1 × 1, 3 × 3, and 5 × 5 convolutions with 1/3 channel allocation, our proposed 4-branch version utilizing 1 × 1, 3 × 3, 5 × 5, and 7 × 7 convolutions with 1/4 channel allocation, and a 5-branch version extending to 1 × 1, 3 × 3, 5 × 5, 7 × 7 and 9 × 9 convolutions with 1/5 channel allocation. All variants were evaluated under identical training conditions, including the same dataset, optimizer settings, and training epochs, to ensure fair comparison.

The experimental results, presented in Table 9, demonstrated that the 4-branch configuration achieved superior performance. The 4-branch MSTA attained an accuracy of 96.11%, precision of 96.17%, recall of 96.11%, and F1-score of 96.14%, representing a significant improvement of 2.04% in accuracy compared to the 2-branch version. While the 3-branch version showed better performance than the 2-branch version, it remained inferior to the 4-branch version. The 5-branch version exhibited performance degradation while increasing computational complexity, which suggested that too many branches may lead to feature redundancy.

The ablation experiment demonstrated that the 4-branch MSTA was the optimal design choice for maize leaf disease identification. The combination of 1 × 1, 3 × 3, 5 × 5, and 7 × 7 convolution kernels with quarter-channel allocation provided sufficient feature representation capacity while avoiding parameter redundancy. This configuration effectively matched the scale distribution characteristics of maize leaf diseases and demonstrated superior generalization capability across different pathological conditions, providing strong support for our architectural design decisions.

#### 3.7.2. Ablation Experiment of DSTANet

To validate the effectiveness of the DSFM and MSTA modules introduced in this study, an ablation experiment was conducted in this section. Based on the baseline MobileViT, the DSFM and the MSTA were added separately, resulting in DSFMViT and MSTAViT. These models, along with MobileViT and DSTANet, were compared.

Table 10 presents the performance of all four models. DSTANet achieved the best overall results with 96.11% accuracy, exceeding DSFMViT (94.85%) by 1.26% and MSTAViT (94.75%) by 1.36%. Both DSFMViT and MSTAViT significantly outperformed MobileViT (90.94%).

To evaluate whether the proposed method could overcome environmental interference and distinguish similar diseases, the model was visualized using Grad-CAM. The results of feature visualization are shown in Table 11. The red region that was identified as key demonstrated how image spatial features correspond to class-specific weights in the classification model.

For MobileViT, some ground and leaf edge regions were shown in red and dark blue, which reflected that MobileViT’s original attention to diseases was diverted, and the complex environment affected the model’s weight calculation for diseases. For example, in the case of PD and ZD, MobileViT failed to effectively differentiate between the ground and weeds, assigning excessive weight to them. Relative to MobileViT, MSTAViT enhanced the perception of lesions by expanding the receptive field for effective feature extraction while simultaneously overcoming the impact of environmental noise. In contrast, DSFMViT excelled at mitigating noise interference, focusing its regions of interest more precisely on diseased areas, which resulted in more accurate and comprehensive localization of pathogenic information. DSTANet combined the advantages of DFSC and MSTA, comprehensively perceived the lesion while accurately extracting the location information of the lesion, and was not affected by noise.

Furthermore, for diseases characterized by spots, such as CR, ES-E, and ES-L, DSTANet disregarded healthy parts of the leaf, intensified its focus on the lesions, and increased the weight assigned to them. For diseases manifesting as stripes, such as GLS and NLB, DSTANet could also effectively and precisely concentrate its attention on the diseased locations.

## 4. Discussion

The architecture effectiveness of DSTANet was validated through 5-fold cross-validation. The consistent performance across all folds (>95% accuracy) demonstrates that the observed high accuracy is not an artifact of favorable data partitioning but rather reflects the inherent effectiveness of our architectural design. This stability across different data splits provides strong evidence for the model’s generalization capability and suggests that the performance improvements achieved by DSTANet are systematic rather than coincidental.

In the comparison with different models, this study comprehensively evaluated DSTANet with seven other mainstream disease identification models. The results showed that DSTANet outperformed all the comparison models in the four key indicators of accuracy, precision, recall, and F1-score. Compared with the optimal model EfficientNetV2 in traditional convolutional neural networks, the accuracy of DSTANet increased by 3.17%. In the comparison with various vision transformer networks, the accuracy rate of DSTANet was also 4.67% higher than that of ViT. These data proved that other models were disturbed to varying degrees by factors such as inter-class similarity, intra-class variability, and complex field environment noise. DSTANet could effectively overcome the above challenges and demonstrate outstanding identification performance. Furthermore, DSTANet’s number of parameters, FLOPs, and inference speed were also superior to those of other models.

In the analysis of similar disease identification, this study visualized the identification results by drawing the confusion matrix. DSTANet could accurately distinguish between gray leaf spot and northern leaf blight, as well as common rust and eyespot. There were only two cases of misclassification for CR and ES, and they had achieved precise identification of GLS and NLB-L. Meanwhile, DSTANet also had significant advantages over other models in the identification of early and late leaf blight and eyespot. As shown in Table 6, the precision of DSTANet for ES-E and NLB-E reached 93.37% and 95.29%, the recall reached 91.50% and 91.00%, and the F1-scores reached 92.42% and 93.10%. This was because DSTANet inherits the advantages of CNN and the transformer.

Ablation experiment of MSTA validated the rationality of 1 × 1, 3 × 3, 5 × 5, and 7 × 7 convolutions with 1/4 channel allocation structures. The 4-branch structure had sufficient feature representation ability and avoided parameter redundancy. Ablation experiments of DSTANet validated the effectiveness of the DSFM and MSTA in this study. As shown in Table 10, models incorporating DSFM or MSTA individually improved accuracy over MobileViT by 3.91% and 3.62%, respectively. DSTANet achieved accuracies 1.26% and 1.36% higher than DSFMViT and MSTAViT, respectively, and 5.17% higher than MobileViT.

Visualization of the identification effects using Grad-CAM showed that the model with the DSFM could effectively concentrate its attention on lesion areas, distinguishing between the background and leaves while assigning different weights to the diseased and healthy regions of the leaves. Furthermore, the model introducing DSFM could capture more detailed texture information and preserve richer feature information from leaf lesions. These findings demonstrate the effectiveness of the DSFM.

MSTAViT demonstrated superior lesion perception capability compared to MobileViT. While MobileViT only focused on partial diseased regions, MSTAViT’s attention endeavored to cover the entire lesion area as comprehensively as possible. These results substantiated that multi-scale token aggregation, as opposed to single-scale convolutional feature extraction methods, could fully integrate information between channel feature maps, thereby enhancing recognition accuracy. DSTANet combined the advantages of DSFM and MSTA, enabling it to not only accurately identify leaf lesion locations while ignoring irrelevant background noise, but also possess stronger feature extraction and information perception capabilities.

However, this study still has the following limitations:

(1) DSTANet lacks the ability to identify multiple labels, and can only recognize the disease with the most prominent symptoms. This limitation may lead to the spread of overlooked diseases, thus missing the best window period for disease prevention and control, increasing the cost of prevention, causing a decline in yield and a deterioration in corn quality, and reducing farmers’ income.
(2) The high accuracy of DSTANet relies on a large amount of training data. However, the data collection of maize disease is subject to various restrictions, and it takes a lot of manpower and material resources to collect enough data.
(3) The dataset we used only covers a few types of maize leaf diseases, and it still needs to be improved to strengthen the generalization ability of the model.

These problems limit the further improvement of the model and still need to be addressed by us. In the future, we will explore a multi-label classification disease recognition algorithm that fuses text and image multi-modal data, and combine generative AI for high-quality data augmentation to improve the performance of the model. And we will deploy the model from this research to mobile devices for farmers’ use as soon as possible, to guide the precise prevention and control of maize diseases and contribute to the sustainable development of the maize industry.

## 5. Conclusions

Taking maize leaf diseases in field environments as the research subject, we created a dataset containing six different categories of maize leaf diseases and healthy leaves. Data for both early and late stages of eyespot and northern leaf blight were also collected. Addressing the challenges brought by inter-class similarity and intra-class variability among leaf diseases, as well as noise interference in the field environment, this study designed DSTANet, which combined CNNs and transformers and introduced the DSFM and MSTA to improve the identification performance of the model. As a result, the DSTANet achieved an accuracy of 96.11%, a precision of 96.17%, a recall of 96.11%, and an F1-score of 96.14%. Furthermore, with a parameter count of only 1.9 M, 0.6GFLOPs, and the capability to recognize 170 images per second, its performance was significantly superior to other models.

In the DSTANet constructed in this study, the introduction of DSFM and MSTA not only simplified the feature extraction process, achieving precise localization of leaf disease lesions, but also significantly enhanced the perception of disease information within the extracted features by fusing multi-scale information. This integration greatly improved the model’s identification efficiency and accuracy. The experimental results demonstrated that DSTANet accurately identified leaf diseases across various types and stages. The introduction of this algorithm enabled users to accurately control the occurrence of diseases in fields, providing technical support for early prevention and control of field diseases, and thereby promoting the precise control and scientific management of maize diseases.

Ablation experiments conducted on the MSTA module and the DSTANet architecture provided support for the rationality of our design choices. The ablation experiment of MSTA showed that the 4-branch MSTA had sufficient feature representation ability and avoided parameter redundancy, which effectively matched the scale distribution characteristics of maize leaf diseases. Ablation experiments of DSTANet also confirmed the effectiveness of DSFM and MSTA, with DSTANet achieving the accuracy of 96.11%, higher 5.17% than the baseline MobileViT.

Compared with other models, DSTANet had significant superiority and could effectively identify early-stage maize diseases, providing guidance for achieving precise prevention and control of early diseases. Future work will focus on model improvement, dataset expansion, and extending this framework to other crop disease identification.

## Figures and Tables

**Table 1:** Data acquisition protocol for maize diseases.

Data Acquisition Protocol	Parameters
Camera model	Xiaomi 13 Ultra (Xiaomi, Beijing, China), Redmi K40 (Xiaomi, Beijing, China)
Camera lens	IMX989 (Sony, Tokyo, Japan), IMX582 (Sony, Tokyo, Japan)
Illumination condition	Sunny, cloudy, rainy, morning, afternoon
Collection Environment	Complex backgrounds (weed, soil, sky, etc.) under natural lighting conditions
Capture Distance	The distance between the leaf and the camera should be within 0.1–0.5 m
Original image resolution	3072 pixels × 4096 pixels
Collection time	From June to August 2024

**Table 2:** Comparison of DSTANet performance for different optimizers.

Optimizer	Epoch	Accuracy	Precision	Recall	F1-Score
SGD	150	91.45%	90.54%	90.86%	90.70%
Adam	150	88.43%	86.43%	87.22%	86.82%
AdamW	150	96.11%	96.17%	96.11%	96.14%

**Table 3:** Comparison of DSTANet performance with different learning rates.

LR	Epoch	Accuracy	Precision	Recall	F1-Score
0.01	150	53.54%	52.25%	43.53%	47.49%
0.001	150	96.11%	96.17%	96.11%	96.14%
0.0001	150	92.03%	91.05%	91.18%	91.11%
0.00001	150	76.58%	73.92%	72.78%	73.35%

**Table 4:** Training parameter setting table.

Parameter	Value
Initial learning rate	1 × 10^−3^
Batch size	64
Epochs	150
Optimizer	AdamW
Lr scheduler	Cosine Annealing
Minimum learning rate	1 × 10^−9^
Loss function	Cross Entropy Loss and Label Smoothing

**Table 5:** DSTANet’s 5-fold cross-validation results.

Fold Number	Accuracy	Precision	Recall	F1-Score
Fold 1	95.56%	96.16%	95.23%	95.69%
Fold 2	95.33%	95.48%	95.32%	95.40%
Fold 3	95.21%	95.64%	94.97%	95.30%
Fold 4	95.46%	94.89%	94.76%	94.82%
Fold 5	95.67%	96.05%	95.25%	95.65%
Average	95.44 ± 0.18%	95.64 ± 0.51%	95.11 ± 0.23%	95.37 ± 0.35%

**Table 6:** Comparison of results of different models.

Model	Accuracy	Precision	Recall	F1-Score
MobileViT	90.94%	91.34%	90.94%	91.14%
DaViT	89.33%	89.84%	89.33%	89.58%
ViT	91.44%	91.73%	91.44%	91.58%
SwinTransformer	91.50%	92.04%	91.49%	91.76%
MobileNetV3	92.39%	92.57%	92.39%	92.48%
EfficientNetV2	92.94%	93.29%	92.94%	93.11%
ConvNeXt	91.72%	92.01%	91.72%	91.86%
VMamba	92.78%	92.94%	92.78%	92.86%
DSTANet	96.11%	96.17%	96.11%	96.14%

**Table 7:** Performance comparison between DSTANet and different models.

Model	Accuracy	Parameters	FLOPs	FPS
MobileViT	90.94%	1.2M	0.41G	155
DaViT	89.33%	28.3M	4.5G	77
ViT	91.44%	86.6M	17.6G	74
SwinTransformer	91.50%	29M	4.5G	124
MobileNetV3	92.39%	2.5M	5.9G	202
EfficientNetV2	92.94%	22M	8.8G	88
ConvNeXt	91.72%	28.6M	4.5G	155
VMamba	92.77%	22M	4.5G	187
DSTANet	96.11%	1.9M	0.6G	170

**Table 8:** The identification results of DSTANet for various diseases.

Type	Precision	Recall	F1-Score
Common Rust (CR)	98.50%	98.50%	98.50%
Gray Leaf Spot (GLS)	98.50%	98.50%	98.50%
Healthy (H)	98.97%	96.50%	97.72%
Early stage Eyespot (ES-E)	93.37%	91.50%	92.42%
Late-stage Eyespot (ES-L)	89.95%	98.50%	94.03%
Early stage Northern Leaf Blight (NLB-E)	95.29%	91.00%	93.10%
Late-stage Northern Leaf Blight (NLB-L)	95.43%	94.00%	94.71%
Phosphorus Deficiency (PD)	99.50%	99.00%	99.25%
Zinc Deficiency (ZD)	96.06%	97.50%	96.77%

**Table 9:** Comparison of MSTA results for different branch versions.

	Kernel Size	Accuracy	Precision	Recall	F1-Score
2-branch	1 × 1, 3 × 3	93.28%	93.32%	93.21%	93.26%
3-branch	1 × 1, 3 × 3, 5 × 5	94.87%	95.12%	94.57%	94.84%
4-branch	1 × 1, 3 × 3, 5 × 5, 7 × 7	96.11%	96.17%	96.11%	96.14%
5-branch	1 × 1, 3 × 3, 5 × 5, 7 × 7, 9 × 9	94.07%	94.02%	94.09%	94.05%

**Table 10:** Effects of DSFM and MSTA on model performance.

Model	DSFM	MSTA	Accuracy	Precision	Recall	F1-Score
MobileViT			90.94%	91.34%	90.94%	91.14%
DSFMViT	√		94.85%	93.88%	93.58%	93.73%
MSTAViT		√	94.56%	93.64%	94.11%	93.87%
DSTANet	√	√	96.11%	96.17%	96.11%	96.14%

**Table 11:** Heat map of ablation experiment.

		MobileViT	MSTAViT	DSFMViT	DSTANet
CR	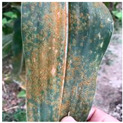	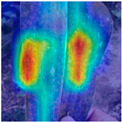	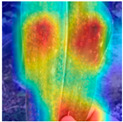	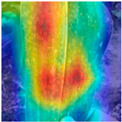	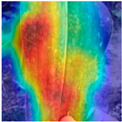
GLS	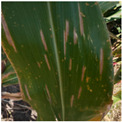	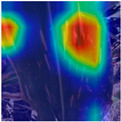	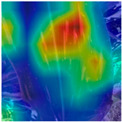	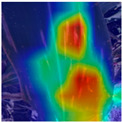	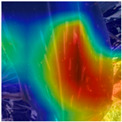
H	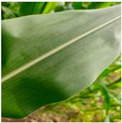	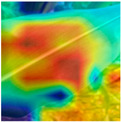	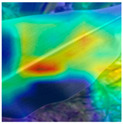	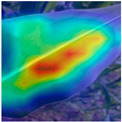	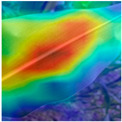
ES-E	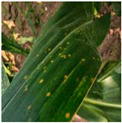	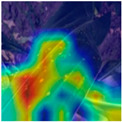	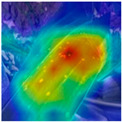	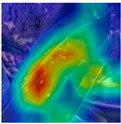	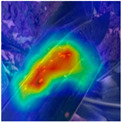
ES-L	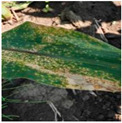	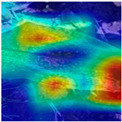	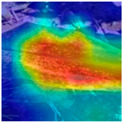	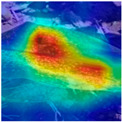	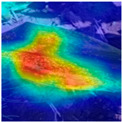
NLB-E	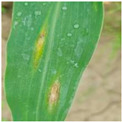	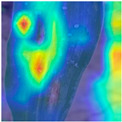	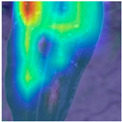	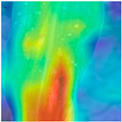	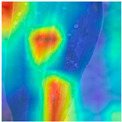
NLB-L	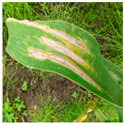	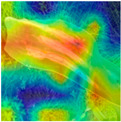	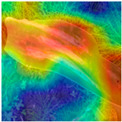	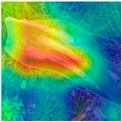	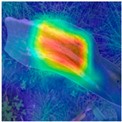
PD	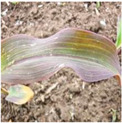	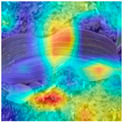	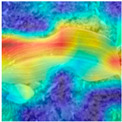	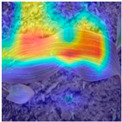	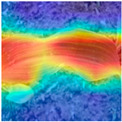
ZD	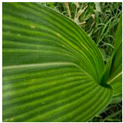	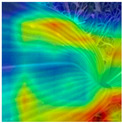	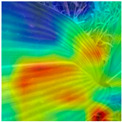	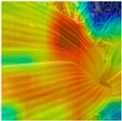	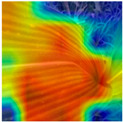

## Data Availability

The data presented in this study are available on request from the corresponding author.
